# Loading Range for the Development of Peak Power in the Close-Grip Bench Press versus the Traditional Bench Press

**DOI:** 10.3390/sports6030097

**Published:** 2018-09-15

**Authors:** Robert G. Lockie, Samuel J. Callaghan, Ashley J. Orjalo, Matthew R. Moreno

**Affiliations:** 1 Department of Kinesiology, California State University, Fullerton, CA 92831, USA; ashley.orjalo@csu.fullerton.edu (A.J.O.); moreno.matthewr@csu.fullerton.edu (M.R.M.); 2 School of Life Sciences, Pharmacy and Chemistry, Kingston University, Kingston upon Thames, KT1 1LQ, UK; S.Callaghan@kingston.ac.uk

**Keywords:** bar mechanics, grip width, linear position transducer, maximal power, optimal load, power profile, upper-body strength

## Abstract

The close-grip bench press (CGBP) is a variation of the traditional bench press (TBP) that uses a narrower grip (~95% biacromial distance) and has application for athletes performing explosive arm actions where the hands are positioned close to the torso. Limited research has investigated CGBP peak power. Twenty-six strength-trained individuals completed a one-repetition maximum TBP and CGBP. During two other sessions, subjects completed two repetitions as explosively as possible with loads from 20% to 90% for each exercise, with peak power measured by a linear position transducer. A factorial ANOVA calculated between- and within-exercise differences in peak power. Partial correlations controlling for sex determined relationships between absolute and relative strength and peak power load. Peak power for the TBP occurred at 50% 1RM, and 30% 1RM for the CGBP. There were no significant (*p* = 0.680) differences between peak power at each load when comparing the TBP and CGBP. For the within-exercise analysis, there were generally no significant differences in TBP and CGBP peak power for the 20–50% 1RM loads. There were no significant relationships between strength and peak power load (*p* = 0.100–0.587). A peak power loading range of 20–50% 1RM for the TBP and CGBP is suggested for strength-trained individuals.

## 1. Introduction

Upper-body strength is a necessity for many athletes [1], and this is commonly demonstrated in pushing actions specific to a particular sport. Some examples of this include a chest pass in basketball and netball [2,3,4], fending in rugby league or rugby union [5,6], and blocking in American football [7]. The bench press is one of the base exercises used to develop upper-body pushing strength [8,9,10]. The traditional bench press (TBP) is performed with a self-selected hand position typically wider than shoulder-width apart, which Young et al. [1] referred to as the “strongest position” for an individual. However, this hand position may not mirror that performed during pushing actions in athletic activities. For example, netball or basketball chest passes are performed with the hands held in front of the chest (i.e., within the shoulders) such that they can grip the ball and push it in a forceful manner to a teammate [2,3,4]. When blocking in American football for offensive linemen (and for other offensive positions), players are coached to ensure that their hands are maintained within the shoulder pads and torso frame of their opponents [7]. This would suggest that, to ensure strength specific to these hand and arm positions, upper-body exercises supplementary to the TBP should also be incorporated in training.

The close-grip bench press (CGBP) is one such exercise. Within the literature, the CGBP has been executed with a grip width on the barbell of 95% biacromial distance (BAD) [11,12,13], which may be closer to that required in the selected pushing movements in sport highlighted earlier [2,3,4,5,6,7]. Within the CGBP exercise itself, the reduction in grip distance generally results in a decrease in the maximum load that can be lifted [12], partially due to changes in the mechanical advantage of the muscles about the shoulder and elbow joints [8,11,14,15]. The CGBP can also cause an increase in the activity of the triceps brachii when compared to the TBP [10]. More notably, previous research has demonstrated that the CGBP can lead to changes in the mechanics (i.e., power, force, and velocity) of this exercise when compared to the TBP [8,12,13].

Understanding the mechanics of the CGBP is important when programming for specific adaptations [10,12,13]. Lockie et al. [12] found that, when compared to a one-repetition maximum (1RM) TBP performed by resistance-trained men and women, the 1RM CGBP led to the generation of greater peak power (376.48 ± 149.66 watts (w) vs. 313.18 ± 105.94 w) and velocity (0.43 ± 0.07 m per second (m·s^−1^) vs. 0.35 ± 0.06 m·s^−1^). Peak power is an important variable to measure and understand, as this is an essential quality for athletes to develop. Training with a load that maximizes peak power could lead to greater power generation at other loads and during other actions (e.g., sport-specific movements such as jumping, throwing, and pushing) [16,17,18,19,20]. Specific to the TBP, Stock et al. [21] discovered that peak power was achieved with 50% of 1RM in strength-trained men. Whether this is also reflected in peak power data for the CGBP has yet to be determined.

In addition, the influence of strength on the load at which peak power is achieved in the CGBP should be derived. Stronger individuals may be more effective coordinating the recruitment of the neuromuscular system, as well as having greater muscle fiber size [22]. What this could mean is that a greater load during a resistance training exercise would be required to achieve peak power in stronger individuals [18,19]. During a bench throw, Baker et al. [18] found that elite rugby league players who had a 1RM TBP of 129.7 ± 14.3 kilograms (kg) achieved peak power with a load which equated to approximately 55% of the 1RM load. These athletes also had a high strength relative to body mass (~1.40 kilograms per kilogram body mass (kg·BM^−1^)). Professional rugby union players, who had a 1RM TBP of 124 ± 19 kg, achieved peak power in a bench throw with a load equivalent to 30% 1RM load [23]. The relative strength (~1.22 kg·BM^−1^) for the athletes analyzed by Bevan et al. [23] was less than that documented by Baker et al. [18], which could have influenced the load at which peak power was attained. It should be determined not only what load peak power may be achieved in the CGBP versus that of the TBP, but whether there is an influence of absolute or relative strength.

Consequently, this study investigated the peak power characteristics of the TBP and CGBP in resistance-trained individuals. All subjects completed a 1RM strength test for both the TBP and CGBP. In separate sessions, subjects also completed the TBP and CGBP lifting a variety of loads (20–90% 1RM) [21,24]. Although this type of profiling has been done for the TBP [21], it has not for the CGBP. A linear position transducer recorded the data for each repetition completed within these sessions. It was hypothesized that the CGBP would feature greater peak power for each load [12]. It was further hypothesized that peak power for both the TBP and CGBP would occur between 30–50% of 1RM for the sample [18,21,23]. Lastly, it was hypothesized that greater absolute and relative strength as measured by the TBP and CGBP would correlate with a higher load at which peak power was attained [18,19].

## 2. Materials and Methods

### 2.1. Subjects

Twenty-six strength-trained individuals (age = 23.58 ± 4.00 years; height = 1.72 ± 0.09 meters (m); body mass = 77.81 ± 16.69 kg), including 20 males (age = 23.85 ± 4.50 years; height = 1.76 ± 0.06 m; body mass = 82.46 ± 15.94 kg) and 6 females (age = 22.67 ± 1.37 years; height = 1.58 ± 0.06 m; body mass = 62.35 ± 7.44 kg), voluntarily participated in this study. Subjects were recruited from the student population at the university. Data were combined for males and females, which has been done in previous strength testing research [12,25,26,27]. This approach to this study was a within-subject analysis, and not any comparisons between the sexes, so this approach was deemed appropriate. Furthermore, Lockie et al. [12] stated that resistance-trained, college-aged males and females (similar to the subjects from this study) can exhibit similar lifting patterns in the TBP and CGBP. All subjects were required to have at least two years of resistance training and bench press experience, and free from any musculoskeletal disorders that would influence their ability to complete the study. G*Power software (v3.1.9.2, Universität Kiel, Düsseldorf, Germany) was used to confirm that the sample size of 26 was sufficient for a repeated measures analysis of variance (ANOVA), within-factors analysis, and ensured the data could be interpreted with a small effect level of 0.30 [28], and a power level of 0.91 when significance was set at 0.05 [29]. For the correlation analysis, data could be interpreted with a moderate effect level of 0.50 [28], and a power level of 0.81 when significance was set at 0.05 [29]. The methodology and procedures used in this study were approved by the California State University, Northridge Committee for the Protection of Human Subjects on 21 March 2016 (1516-167), and conformed to the policy statement with respect to the Declaration of Helsinki. All subjects received a clear explanation of the study, including the risks and benefits of participation, and written informed consent was obtained prior to testing.

### 2.2. Procedures

Three testing sessions were used for this study, with a minimum of 48 h between sessions. Prior to data collection in the first session, each subject’s age, height, body mass, and BAD were recorded. Height was measured barefoot using a portable stadiometer (Seca, Hamburg, Germany). Body mass was recorded by electronic digital scales (Tanita Corporation, Tokyo, Japan). BAD was determined by palpating and marking the acromion with a makeup pencil, and then measuring the distance between these points with a standard anthropometric measuring tape (Lufkin, Cleveland, OH, USA) [15]. In the first session, subjects were assessed in their 1RM TBP and 1RM CGBP via the procedures detailed below. The exercise completed first was randomized amongst the sample via the randomization function in a Microsoft Excel spreadsheet (Microsoft Corporation, Redmond, WA, USA) [12,13]. In Sessions 2 and 3, subjects completed either the TBP or CGBP. For each exercise, two repetitions were completed at loads ranging from 20% to 90% (at 10% increments) of the 1RM for the respective lift. The exercise completed in each session was randomized amongst the sample using the randomization function in a Microsoft Excel spreadsheet, and the loads were completed in an ascending order [21]. Subjects were tested on the same time of day for both testing sessions, refrained from intensive upper-body exercise and any form of stimulant in the day prior to testing, and maintained a standardized dietary intake in the 24-h period prior to testing [12,13,26,30,31,32,33]. No bench press suits, weightlifting belts, or other supportive garments were permitted. 

### 2.3. 1RM TBP and CGBP Strength Testing

The 1RM testing methods were based upon previous research [12,13]. Although the procedures have been documented by Lockie et al. [12] and Lockie et al. [13], they are presented here. An Olympic bar and weight plates (American Barbell, San Diego, CA, USA) were used for both bench press lifts. The descriptions here detail the process if the TBP was performed first. To complete the TBP, subjects laid recumbent on a flat bench with their feet contacting the floor, and their head, shoulders and buttocks flat to the bench. Subjects self-selected the hand position for the TBP, used a pronated grip, and were told to select their “strongest position” for the grip width [1]. The distance between the index fingers was measured, such that this could be made relative to the subject’s BAD. The distance was marked on the barbell with strips of athletic tape to certify subjects placed their hands on the bar at the same position for each repetition. The subject unracked the bar, and began the lift with the arms extended [8,9,12,13]. The “touch-and-go” procedure was utilized, where the bar was required to touch the chest before being pressed to full arm extension [34]. A successful repetition was attained when the bar was lifted from the chest to the position of full elbow extension [35]. Failure to do this, or having the bar bounce on the chest, disqualified a repetition. Subjects were also required to ensure their head, shoulders, and hips remained in contact with the bench, while both feet remained in contact with the floor. A spotter was positioned behind the bar for assistance with lift-off if required and for safety, but was not to touch the bar except in the event of a failed lift [15]. The procedures for the warm-up to the 1RM were adapted from Stock et al. [21], with between-set recovery periods of 2 min provided. Subjects began by completing 8–10 repetitions at 50% of their estimated 1RM, followed by 3–5 repetitions at 85% estimated 1RM. Subjects then completed one repetition with 90% of the estimated 1RM, before attempting their first attempt at the 1RM. If successful, loads would increase by 2.5-kg increments until the subject failed to complete a repetition. No more than five attempts were needed before the 1RM was reached for all subjects in this study. Three minutes of recovery was provided between attempts.

After a 10 min recovery period, subjects then completed the CGBP [12,13]. The body position and constraints that determined a successful lift were the same as that for the TBP, except for the different grip width. The grip width adopted for the CGBP was 95% of BAD [11,12,13]; this placed the hands in a position within the shoulders. This hand position is comparable to that required in upper-body pushing motions in sports [2,3,4,7], and therefore was adopted in the present research. The grip width was marked on the barbell with athletic tape, and a pronated grip was again utilized. Following established procedures [8,12,13], the warm-up for the second strength test began by completing 3–5 repetitions at 85% of the subjects’ estimated 1RM, and then one repetition with 90% 1RM. Subjects then attempted their first 1RM attempt following a 3-min recovery period, and this process continued until the 1RM was reached. For both the TBP and CGBP, absolute strength was taken as the maximum load lifted. Relative strength was calculated according to the equation: relative strength (kg·BM^−1^) = 1RM TBP or CGBP∙BM^−1^. The absolute and relative strength measures were used for analysis of the influence of strength on the load in which peak power was achieved.

### 2.4. TBP and CGBP Peak Power Testing

Two testing sessions were used for the experimental trials. As stated, in one session, subjects completed the TBP, and in the other they completed the CGBP. Both the TBP and CGBP was completed as described for the 1RM testing. The grip width used for the TBP was the preferred distance subjects self-selected during the 1RM strength test, while the grip width for the CGBP was 95% of BAD (i.e., the same grip width from the 1RM CGBP test). These were marked on the barbell with a thin strips of athletic tape at the start of each session, prior to subjects completing a warm-up consisting of 2–4 sets of the specific exercise (i.e., the TBP of CGBP) with a submaximal, light load (e.g., <40% 1RM) for 6–10 repetitions [21,36]. Following a 2-min rest period, subjects completed two repetitions [36,37] with loads equating to 20%, 30%, 40%, 50%, 60%, 70%, 80%, and 90% of TBP or CGBP 1RM. Following the procedures of Stock et al. [21], the loads were completed in sequential order. The actual weight lifted was within ±1 kg of that calculated from the 1RM [36]. At each load, subjects were told to lower the bar with control, before completing the concentric phase as forcefully and rapidly as possible to maximize power output [21,36]. Recovery periods of 2–3 min were provided between each load [21,36].

Data was recorded during each TBP and CGBP repetition by a GymAware Powertool linear position transducer (Kinetic Performance Technology, Canberra, Australia). The GymAware Powertool features a spring-loaded retractable cable that passes around a spool integrated with an optical encoder [38], and the external end of the cable was attached on the inside of the barbell (i.e., inside the plates, and on the outer part of the grip section of the bar) [12,13,26,31]. The GymAware Powertool was then placed on the floor directly underneath the bar, with the magnetic bottom placed on top of a weight plate to ensure the unit did not move during each lift. The encoder recorded velocity and the movement of the bar; barbell mass was entered into the software to calculate force and power output, for every 3 mm of bar movement [38]. The cable provided no additional resistance to the bar, and the GymAware Powertool has been shown to produce reliable and valid data when measuring concentric power [16,39,40]. Peak power during the concentric phase of the lift for each repetition was collected and stored on an iPad handheld device (Apple Inc., Cupertino, CA, USA), before being uploaded to an online database. The data were then extracted from this database and entered into Microsoft Excel (Microsoft Corporation, Redmond, WA, USA) prior to statistical analyses. The best repetition, defined by the repetition with the highest peak power value, was used for analysis.

### 2.5. Statistical Analysis

All statistics were computed using the Statistics Package for Social Sciences Version 22.0 (IBM, Armonk, NY, USA). Descriptive statistics (mean ± standard deviation [SD]) were used to profile each measured parameter. A 2 × 8 (bench press exercise (TBP and CGBP) × load (20%, 30%, 40%, 50%, 60%, 70%, 80%, and 90% 1RM)) within factorial ANOVA was conducted [21]. Mauchly’s test of sphericity was checked, and the Greenhouse–Geisser correction was applied if sphericity was violated. Significance for the factorial ANOVA was set at *p* < 0.05. Significant main effects of load were investigated further via post hoc analysis using a Bonferroni adjustment for multiple pairwise comparisons. Partial correlations (*r*) controlling for sex were calculated with respect to absolute and relative strength as measured by the 1RM TBP and CGBP, and the load at which peak power occurred. Significance was set as *p* < 0.05 a priori.

## 3. Results

**Figure 1:**
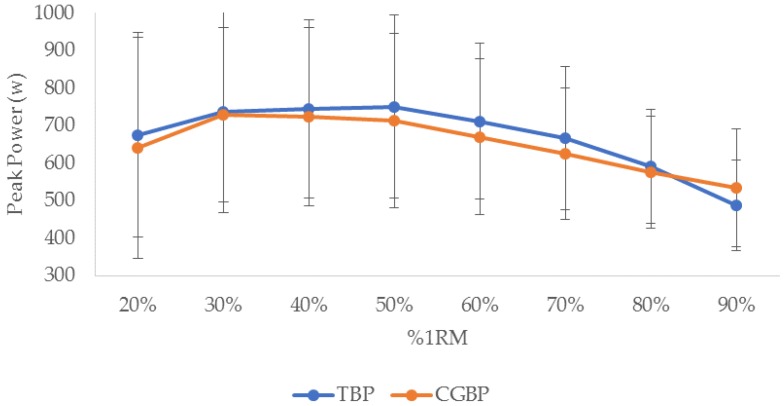
Peak power (mean ± SD) achieved using different loads (20–90% 1RM) in the traditional bench press (TBP and close-grip bench press (CGBP) in strength-trained individuals (*n* = 26).

The mean grip width for the TBP was 0.61 ± 0.12 m, while for the CGBP it was 0.34 ± 0.04 m. Figure 1 displays the power curves for the TBP and CGBP. Peak power for the TBP occurred at 50% 1RM (750.15 ± 261.02 w) while peak power for the CGBP occurred at 30% 1RM (727.55 ± 251.49 w). The results of the 2 × 8 within factorial ANOVA indicated that the interaction between exercise type and power was not significant (*p* = 0.680). Due to this finding, independent repeated measures ANOVA with Bonferroni post-hoc were used to examine the within-exercise differences between loads for the TBP and CGBP. Table 1 displays the within-exercise peak power comparisons for the TBP. There were no significant differences in peak power between the 20–60% 1RM loads. The peak power at 50% 1RM was significantly greater than the 70% 1RM load. Peak power at 80% 1RM was significantly lower than loads at 30–70% 1RM, while peak power at 90% 1RM was significantly lower than all other loads.

Table 2 shows the within-exercise peak power data for the CGBP. There were no significant differences in peak power for the 20–50% 1RM loads. The peak power at 30% and 40% 1RM was significantly greater than the 60% and 70% 1RM loads. Peak power at 50% 1RM was also significantly greater than the 70% 1RM load. Peak power at 80% 1RM was significantly lower than loads at 30–60% 1RM, while peak power at 90% 1RM was significantly lower than loads at 30–70% 1RM.

The mean 1RM for the TBP was 86.43 ± 27.95 kg, with relative strength equaling 1.15 ± 0.23 kg·BM^−1^. The 1RM for the CGBP was 82.23 ± 25.35 kg, with relative strength equating to 0.84 ± 0.50 kg·BM^−1^. There were no significant correlations between absolute (*r* = −0.139 *p* = 0.507) and relative (*r* = −0.116, *p* = 0.580) strength to the load at which peak power occurred for the TBP. There were also no significant correlations for the CGBP (absolute: *r* = −0.336, *p* = 0.100; relative: *r* = −0.330, *p* = 0.107).

## 4. Discussion

This is the first study to investigate the peak power for the CGBP with comparisons to the TBP. The grip widths for the TBP and CGBP in this study were consistent with previous research, which has indicated these distances are significantly different in strength-trained men and women [12,13]. Peak power tended to occur within the same range for both the TBP and CGBP, which was 20–50% 1RM. This is similar to previous research in bench press exercises [18,21,23]. There were no significant differences between peak power at each load when comparing the TBP and CGBP, which was contrary to the studies hypotheses. Additionally, absolute and relative strength did not appear to relate to when load peak power occurred for strength-trained men and women. The findings from this study have useful applications for strength and conditioning practitioners.

Peak power for the TBP occurred at 50% 1RM for the subjects in this study, while for the CGBP it was 30% 1RM. These loads relate well to established standards from the literature. Stock et al. [21] found that peak power was achieved with 50% 1RM for the bench press in strength-trained men, while Baker et al. [18] illustrated that elite rugby league players generated peak power in the bench throw at 55% 1RM. Bevan et al. [23] found that professional rugby union players generated peak power at 30% 1RM in the bench throw. There were, however, some differences in the data from this study with previous research. Stock et al. [21] found significant increases in peak power from 10% 1RM to 50% 1RM, followed by significant decreases through to 90% 1RM. This was somewhat different to the results from this research. Although peak power occurred for the TBP at 50% 1RM and for the CGBP at 30% 1RM, the power generated was generally not significantly different for 20–50% 1RM. However, peak power at the heaviest loads of 80–90% 1RM was generally lower than that for all other loads.

There are some important implications from these results. When training to develop peak power in an upper-body pushing exercise, it is important to select a load that maximizes this output [20,23,24]. In strength-trained men and women for both the TBP and CGBP, a loading range of 20–50% 1RM could be used to optimize peak power development. This is beneficial information for the practitioner, as these data provide a practical range to utilize for both the TBP and CGBP. No previous study has provided these data for the CGBP, which is important given the potential movement specificity of this exercise relative to the TBP [2,3,4,5,6,7,12,13].

Interestingly, there were no significant differences between the peak power generated at each load when comparing between the TBP and CGBP. As Lockie et al. [12] found that greater power was generated in the 1RM CGBP compared to the TBP in resistance-trained men and women, it was expected that this could also occur at lighter loads; however, this was not the case. Lockie et al. [13] did find significant correlations between the power generated in the TBP and CGBP, which could be expected within an individual (i.e., an individual that can generate high peak power in the TBP should be able to do so in the CGBP). Nonetheless, there are practical applications for these findings. As noted, the hand position for the CGBP more closely mirrors that required in sports such as basketball and netball [2,3,4], rugby league and union [5,6], and American football [7]. As TBP peak power can improve with the consistent application of pressing exercises [41], the CGBP could be used to generate peak power values similar to that for the TBP, while utilizing an upper-body position more specific to sports [2,3,4,5,6,7,12,13]. Future research should investigate whether CGBP peak power can improve with consistent training in strength-trained and athletic populations.

There were no significant relationships between absolute and relative strength for the TBP and CGBP, and the load at which peak power occurred. This was contrary to the studies hypothesis, in addition to previous research [18,19]. A potential reason for this discrepancy may have been the strength of the subjects in the current research versus those from Baker et al. [18]. The subjects from this study had a 1RM for the TBP of 86.43 ± 27.95 kg, and 82.23 ± 25.35 kg for the CGBP, which equated to relative strength values of 1.15 ± 0.23 kg·BM^−1^ and 0.84 ± 0.50 kg·BM^−1^, respectively. These values were well below those recorded by Baker et al. [18] (absolute strength = 129.7 ± 14.3 kg; relative strength = ~1.40 kg·BM^−1^). If stronger subjects were analyzed in the TBP and CGBP, they may generate peak power at a higher load than those in this study. Nevertheless, the peak power loading range for both the TBP and CGBP in this study was consistent with established standards [18,21,23]. In accordance with this, for strength-trained individuals who are not high-level athletes, peak power in the TBP and CGBP may be less influenced by absolute or relative strength, and should typically be generated between 20% and 50% 1RM.

There are certain study limitations that should be acknowledged. A biomechanical analysis of the lifting technique for the TBP and CGBP was not conducted, so it is not known whether there were differences in how peak power was achieved in the TBP and CGBP across the loads, and how this could influence potential training adaptations. Further, electromyography was also not recorded in this study. Muscle recruitment patterns can differ between the TBP and CGBP [10,14,15], and this may influence how peak power is generated and any adaptations that could result. As noted, while the subjects in this study were strength-trained, they were not as strong as high-level athletes [18]. Future research should investigate peak power in the CGBP from stronger individuals to document whether different results occur to that from the current study.

## 5. Conclusions

This study documented that peak power in the CGBP was similar to that for the TBP across loads from 20% to 90% 1RM. For the subjects in this study, peak power in the TBP occurred at 50% 1RM, while for the CGBP it occurred at 30% 1RM. However, for both bench press exercises, a peak power loading range of 20–50% 1RM can be suggested as there were no significant differences between these loads. Additionally, absolute and relative strength did not significantly correlate with the load at which peak power occurred for either the TBP or CGBP. This may have been influenced by the strength of the subjects analyzed in this study. Nevertheless, the aforementioned loading ranges were consistent with previous research. To reiterate, for strength-trained men and women, a loading range of 20–50% 1RM for both the TBP and CGBP is suggested for the generation of peak power.

## Figures and Tables

**Table 1:** Within-exercise comparisons between the peak power generated at each load for the TBP in strength-trained individuals (*n* = 26).

%1RM	20%	30%	40%	50%	60%	70%	80%
30%	0.783	-	-	-	-	-	-
40%	0.240	1.000	-	-	-	-	-
50%	0.501	1.000	1.000	-	-	-	-
60%	1.000	1.000	1.000	0.835	-	-	-
70%	1.000	1.000	0.080	0.001 *	0.702	-	-
80%	0.894	0.009 *	<0.001 *	<0.001 *	<0.001 *	<0.001 *	-
90%	0.001 *	<0.001 *	<0.001 *	<0.001 *	<0.001 *	<0.001 *	0.002 *

* Significant (*p* < 0.05) difference between the two loads.

**Table 2:** Within-exercise comparisons between the peak power generated at each load for the CGBP in strength-trained individuals (*n* = 26).

%1RM	20%	30%	40%	50%	60%	70%	80%
30%	0.293	-	-	-	-	-	-
40%	0.635	1.000	-	-	-	-	-
50%	1.000	1.000	1.000	-	-	-	-
60%	1.000	0.006 *	0.016 *	0.079	-	-	-
70%	1.000	0.002 *	0.004 *	0.009 *	0.123	-	-
80%	1.000	<0.001 *	<0.001 *	<0.001 *	0.003 *	0.107	-
90%	1.000	<0.001 *	<0.001 *	<0.001 *	0.002 *	0.014 *	0.109

* Significant (*p* < 0.05) difference between the two loads.

## Abbreviations

The following abbreviations are used in this manuscript:

TBP	Traditional bench press
CGBP	Close-grip bench press
BAD	Biacromial distance
1RM	One-repetition maximum
w	Watts
kg	Kilograms
kg·BM^−1^	Kilograms per kilogram body mass
BM	Body mass
m	Meters
ANOVA	Analysis of variance
SD	Standard deviation
*p*	Significance
*r*	Correlation coefficient

## References

[1] Young K.P., Haff G.G., Newton R.U., Gabbett T.J., Sheppard J.M. (2015). Assessment and monitoring of ballistic and maximal upper-body strength qualities in athletes. Int. J. Sports Physiol. Perform..

[2] Cronin J.B., Owen G.J. (2004). Upper-body strength and power assessment in women using a chest pass. J. Strength Cond Res..

[3] Delextrat A., Cohen D. (2009). Strength, power, speed, and agility of women basketball players according to playing position. J. Strength Cond Res..

[4] Hoare D.G. (2000). Predicting success in junior elite basketball players—The contribution of anthropometic and physiological attributes. J. Sci. Med. Sport.

[5] Wheeler K., Sayers M. (2009). Contact skills predicting tackle-breaks in rugby union. Int. J. Sports Sci. Coach..

[6] Wheeler K.W., Sayers M.G.L. (2011). Rugby union contact skills alter evasive agility performance during attacking ball carries. Int. J. Sports Sci. Coach..

[7] Stokes J.V., Luiselli J.K., Reed D.D., Fleming R.K. (2010). Behavioral coaching to improve offensive line pass-blocking skills of high school football athletes. J. Appl. Behav. Anal..

[8] Gomo O., Van Den Tillaar R. (2016). The effects of grip width on sticking region in bench press. J. Sports Sci..

[9] Algra B. (1982). In-depth analysis of the bench press. Natl. Strength Cond. Assoc. J..

[10] Lehman G.J. (2005). The influence of grip width and forearm pronation/supination on upper-body myoelectric activity during the flat bench press. J. Strength Cond. Res..

[11] Wagner L.L., Evans S.A., Weir J.P., Housh T.J., Johnson G.O. (1992). The effect of grip width on bench press performance. Int. J. Sport Biomech..

[12] Lockie R.G., Callaghan S.J., Moreno M.R., Risso F.G., Liu T.M., Stage A.A., Birmingham-Babauta S.A., Stokes J.J., Giuliano D.V., Lazar A. (2017). An investigation of the mechanics and sticking region of a one-repetition maximum close-grip bench press versus the traditional bench press. Sports.

[13] Lockie R.G., Callaghan S.J., Moreno M.R., Risso F.G., Liu T.M., Stage A.A., Birmingham-Babauta S.A., Stokes J.J., Giuliano D.V., Lazar A. (2017). Relationships between mechanical variables in the traditional and close-grip bench press. J. Hum. Kinet..

[14] Barnett C., Kippers V., Turner P. (1995). Effects of variations of the bench press exercise on the EMG activity of five shoulder muscles. J. Strength Cond. Res..

[15] Clemons J.M., Aaron C. (1997). Effect of grip width on the myoelectric activity of the prime movers in the bench press. J. Strength Cond. Res..

[16] Argus C.K., Gill N.D., Keogh J.W., Hopkins W.G. (2011). Assessing lower-body peak power in elite rugby-union players. J. Strength Cond. Res..

[17] Turner A.P., Unholz C.N., Potts N., Coleman S.G. (2012). Peak power, force, and velocity during jump squats in professional rugby players. J. Strength Cond. Res..

[18] Baker D., Nance S., Moore M. (2001). The load that maximizes the average mechanical power output during explosive bench press throws in highly trained athletes. J. Strength Cond. Res..

[19] Baker D., Nance S., Moore M. (2001). The load that maximizes the average mechanical power output during jump squats in power-trained athletes. J. Strength Cond. Res..

[20] Castillo F., Valverde T., Morales A., Pérez-Guerra A., de León F., García-Manso J.M. (2012). Maximum power, optimal load and optimal power spectrum for power training in upper-body (bench press): A review. Rev. Andal. Med. Deporte.

[21] Stock M.S., Beck T.W., Defreitas J.M., Dillon M.A. (2010). Relationships among peak power output, peak bar velocity, and mechanomyographic amplitude during the free-weight bench press exercise. J. Sports Sci..

[22] Folland J.P., Williams A.G. (2007). The adaptations to strength training: Morphological and neurological contributions to increased strength. Sports Med..

[23] Bevan H.R., Bunce P.J., Owen N.J., Bennett M.A., Cook C.J., Cunningham D.J., Newton R.U., Kilduff L.P. (2010). Optimal loading for the development of peak power output in professional rugby players. J. Strength Cond. Res..

[24] Billich R., Štvrtňa J., Jelen K. (2014). Optimal velocity to achieve maximum power output—Bench press for trained footballers. AUC Kinanthropol..

[25] Berning J.M., Coker C.A., Briggs D. (2008). The biomechanical and perceptual influence of chain resistance on the performance of the olympic clean. J. Strength Cond. Res..

[26] Lockie R.G., Moreno M.R., Lazar A., Risso F.G., Tomita T.M., Stage A.A., Birmingham-Babauta S.A., Torne I.A., Stokes J.J., Giuliano D.V. (2018). The 1-repetition maximum mechanics of a high-handle hexagonal bar deadlift compared to a conventional deadlift as measured by a linear position transducer. J. Strength Cond. Res..

[27] Thompson B.J., Stock M.S., Shields J.E., Luera M.J., Munayer I.K., Mota J.A., Carrillo E.C., Olinghouse K.D. (2015). Barbell deadlift training increases the rate of torque development and vertical jump performance in novices. J. Strength Cond. Res..

[28] Hopkins W.G. (2004). How to interpret changes in an athletic performance test. Sportscience.

[29] Faul F., Erdfelder E., Lang A.G., Buchner A. (2007). G*Power 3: A flexible statistical power analysis program for the social, behavioral, and biomedical sciences. Behav. Res. Methods.

[30] Lockie R.G., Callaghan S.J., Berry S.P., Cooke E.R., Jordan C.A., Luczo T.M., Jeffriess M.D. (2014). Relationship between unilateral jumping ability and asymmetry on multidirectional speed in team-sport athletes. J. Strength Cond. Res..

[31] Lockie R.G., Moreno M.R., Orjalo A.J., Lazar A., Liu T.M., Stage A.A., Birmingham-Babauta S.A., Stokes J.J., Giuliano D.V., Risso F.G. (2017). The relationships between height, arm length, and leg length on the mechanics of the conventional and high-handle hexagonal bar deadlift. J. Strength Cond. Res..

[32] Lockie R.G., Schultz A.B., Jordan C.A., Callaghan S.J., Jeffriess M.D., Luczo T.M. (2015). Can selected functional movement screen assessments be used to identify movement deficiencies that could affect multidirectional speed and jump performance?. J. Strength Cond. Res..

[33] Lockie R.G., Schultz A.B., Callaghan S.J., Jordan C.A., Luczo T.M., Jeffriess M.D. (2015). A preliminary investigation into the relationship between functional movement screen scores and athletic physical performance in female team sport athletes. Biol. Sport.

[34] Ware J.S., Clemens C.T., Mayhew J.L., Johnston T.J. (1995). Muscular endurance repetitions to predict bench press and squat strength in college football players. J. Strength Cond. Res..

[35] Robbins D.W., Young W.B., Behm D.G. (2010). The effect of an upper-body agonist-antagonist resistance training protocol on volume load and efficiency. J. Strength Cond. Res..

[36] Swinton P.A., Stewart A., Agouris I., Keogh J.W., Lloyd R. (2011). A biomechanical analysis of straight and hexagonal barbell deadlifts using submaximal loads. J. Strength Cond. Res..

[37] Bosquet L., Porta-Benache J., Blais J. (2010). Validity of a commercial linear encoder to estimate bench press 1 RM from the force-velocity relationship. J. Sports Sci. Med..

[38] Drinkwater E.J., Moore N.R., Bird S.P. (2012). Effects of changing from full range of motion to partial range of motion on squat kinetics. J. Strength Cond. Res..

[39] Drinkwater E.J., Galna B., McKenna M.J., Hunt P.H., Pyne D.B. (2007). Validation of an optical encoder during free weight resistance movements and analysis of bench press sticking point power during fatigue. J. Strength Cond. Res..

[40] Ball N., Nolan E., Wheeler K. (2011). Anthropometrical, physiological, and tracked power profiles of elite taekwondo athletes 9 weeks before the Olympic competition phase. J. Strength Cond. Res..

[41] Baker D.G. (2013). 10-year changes in upper body strength and power in elite professional rugby league players—The effect of training age, stage, and content. J. Strength Cond. Res..

